# Latent Class Analysis of Obesogenic Behaviors among Korean Adolescents: Associations with Weight-Related Outcomes

**DOI:** 10.3390/ijerph182111059

**Published:** 2021-10-21

**Authors:** Haein Lee, In-Seo La

**Affiliations:** 1 College of Nursing, Research Institute of Nursing Science, Daegu Catholic University, Daegu 42472, Korea; hlee1317@cu.ac.kr; 2 College of Nursing Science, Kyung Hee University, Seoul 02447, Korea

**Keywords:** adolescent, obesogenic behaviors, obesity, unhealthy weight control behaviors, latent class analysis

## Abstract

This study aimed to explore sex-specific latent class models of adolescent obesogenic behaviors (OBs), predictors of latent class membership (LCM), and associations between LCM and weight-related outcomes (i.e., weight status and unhealthy weight control behaviors). We analyzed nationally representative data from the 2019 Korea Youth Risk Behavior Survey. To identify latent classes for boys (*n* = 29,841) and girls (*n* = 27,462), we conducted a multiple-group latent class analysis using eight OBs (e.g., breakfast skipping, physical activity, and tobacco product use). Moreover, we performed a multinomial logistic regression analysis and a three-step method to examine associations of LCM with predictors and weight-related outcomes. Among both sexes, the 3-class models best fit the data: (a) mostly healthy behavior class, (b) poor dietary habits and high Internet use class, and (c) poor dietary habits and substance use class. School year, residential area, academic performance, and psychological status predicted the LCM for both sexes. In addition, perceived economic status predicted the LCM for girls. The distribution of weight-related outcomes differed across sex-specific classes. Our findings highlight the importance of developing obesity prevention and treatment interventions tailored to each homogeneous pattern of adolescent OBs, considering differences in their associations with predictors and weight-related outcomes.

## 1. Introduction

Adolescence is one of the critical development periods, with an increasing risk of obesity [1]. The increase in adolescent obesity has become a global public health concern [2]. For example, obesity prevalence in U.S. adolescents increased from 18.1% in 2007–2008 to 21.2% in 2017–2018 [3]. Researchers have reported on a marked increase in the prevalence of adolescents with obesity in East Asia [2]. Indeed, the prevalence of obesity in Korean adolescents has doubled from 5.3% in 2007 to 10.8% in 2018 [4]. Adolescent obesity causes physical (e.g., hypertension, insulin resistance, and gastrointestinal disease) and mental (e.g., depression, anxiety, and eating disorder) problems, besides increasing healthcare costs [5,6]. Furthermore, adolescents with obesity are more likely to be obese in adulthood, with greater risks of cardiovascular disease, type 2 diabetes, and cancer [7]. 

The prevention of adolescent obesity-related problems necessitates special attention to unhealthy weight-control behaviors (UWCBs) as well as weight status [8]. UWCBs (e.g., fasting, skipping meals, using weight-loss pills, and vomiting) are prevalent among adolescents [9]. For example, the prevalence of UWCBs in U.S. and Korean adolescents range from 13.6% to 15.0% [10,11]. In addition, the literature indicated that adolescents’ UWCBs are associated with increased body mass index (BMI), eating disorders, depression, and suicidal behaviors [9,11,12]. 

Considering the high rates and consequences of adolescent obesity and UWCB, it is imperative to develop effective strategies to prevent and treat weight-related problems. The theoretical framework guiding Project EAT provides the basis for the importance of obesogenic behaviors (OBs) for improving weight-related outcomes [13]. Adolescents’ OBs (e.g., poor dietary habits, low physical activities, sedentary behaviors, and substance use) are associated with their weight status and UWCB [14,15,16]. Furthermore, these OBs occur concurrently and interact with each other [17], besides exerting combined effects on obesity-related health [18]. Considering the limitations of single-dimensional approaches (e.g., focusing on a single OB or the number of OBs) in explaining the complexity and multidimensionality of OBs, it is necessary to identify patterns of multiple OBs and develop differential interventions reflecting the characteristics of each pattern [18].

Thus, recent researchers have identified latent classes or clusters of adolescent OBs and have investigated their associations with weight-related outcomes [18,19,20]. In particular, latent class analysis (LCA), one of the person-centered approaches, is considered suitable for (a) identifying heterogeneous subgroups based on multidimensional characteristics of human behaviors [21] and (b) examining effects of tailored interventions considering characteristics of target subgroups [22]. Most LCA studies identifying patterns of adolescent OBs demonstrated that multiple OBs were divided into three to five heterogeneous subgroups [19], associated with weight status, perceived overweight, and body dissatisfaction [19,20,23,24,25]. However, there is little information available on the association between OB patterns and UWCB. Given the prevalence and detrimental consequences of UWCB [8,9,11,12], further investigation on the association between OB patterns and UWCB among adolescents is necessary.

Furthermore, previous studies examining patterns of adolescent OBs are limited in two aspects. First, the majority of the existing LCA studies have limited information on sex differences in OBs [19]. It is necessary to identify sex-specific OB patterns, considering sex differences in exposure levels and the vulnerability to obesogenic environments, weight-related outcomes, and responses to obesity interventions [26]. Second, despite adolescent OBs being affected by cultural characteristics [19], all LCA studies investigating OB patterns have been conducted in Western countries [19,20,24]. Thus, this study aimed to explore sex-specific patterns of OBs among Korean adolescents, to identify predictors of latent class membership (LCM), and to investigate if distinct OB classes exert differential effects on weight status and UWCB.

## 2. Materials and Methods

### 2.1. Data and Participants

We used data acquired from the 15th Korea Youth Risk Behavior Survey (KYRBS) conducted in 2019. This web-based survey is conducted annually to identify health behaviors such as dietary behavior, physical activity, and substance use of Korean adolescents [27]. The 15th KYRBS used a stratified multi-stage cluster sample design to collect a nationally representative sample of 7th–12th grade students. The sample comprised 57,303 students from 400 middle schools and 400 high schools [27]. Student age ranged from 12 to 18 years old (mean = 15.08, standard deviation = 1.78). We obtained an exemption from an institutional review board prior to the initiation of this study because it used a de-identified data set (IRB No. CUIRB-2021-E008). 

### 2.2. Measures

#### 2.2.1. Obesogenic Behaviors

To estimate sex-specific patterns of adolescent OBs, we selected and dichotomized eight indicators based on the literature [15,16,19,20,28]. The questionnaire consisted of three parts: dietary behavior, physical activity and sedentary behavior, and substance use. First, we assessed four dietary behaviors by inquiring about the following eating habits during the past seven days: (a) >2 days of breakfast skipping [29], (b) ≥3 days of sugar-sweetened beverage (e.g., soft drinks, carbonated drinks, juice or flavored drinks; SSB) intake [24], (c) ≥3 days of fast food consumption [15], and (d) non-daily fruit and vegetable consumption [30]. Second, we used two questions regarding their total physical activity and non-academic Internet use (NAIU) to assess their physical activity and sedentary behavior. Total physical activity was assessed by inquiring if the participants had engaged in any physical activity that increased their heart rate and caused a shortness of breath for at least 60 min each day during the past seven days [28]. To assess NAIU, we investigated the average hours of using the Internet for non-academic purposes on weekdays and weekends. We calculated the average daily hours spent in NAIU using the following formula: (5 × the average NAIU hours on weekdays + 2 × the average NAIU hours on weekends)/7. The responses were classified into “≥2 h” and “<2 h” per day [16]. Third, we assessed substance use by asking the following: (a) monthly tobacco product use (i.e., using at least one of cigarette, electronic cigarette, or heated tobacco product in the past 30 days) and (b) their monthly alcohol use (i.e., alcohol consumption at least once in the past 30 days). 

#### 2.2.2. Predictors of LCM

To identify the predictors associated with LCM, we selected factors based on the literature [18,31,32,33,34,35,36]. We included demographics (i.e., school year, area of residence, and perceived economic status), academic performance, and psychological status (i.e., stress, depressive feeling, and sleep satisfaction). School year was classified into “middle school” and “high school.” Area of residence was divided into “suburban or rural area” and “urban area.” Perceived economic status and academic performance were assessed using a 5-point Likert scale, ranging from “very high” (0) to “very low” (4). We dichotomized the responses into two categories: “high or middle” and “low.” 

Stress was assessed by asking participants about their usual level of stress. Possible responses ranged from “very low” (0) to “very high” (4) and were categorized into “low or average” and “high.” Depressive feeling was assessed by questioning if the participants had experienced considerable sadness or despair to interrupt their daily activities in the past two weeks. The possible responses were “yes” and “no.” We measured sleep satisfaction by inquiring how satisfied they were with their sleep to relieve fatigue during the past seven days. Possible responses ranged from “very satisfied” (0) to “very dissatisfied” (4) and were categorized into “satisfied or average” and “dissatisfied.”

#### 2.2.3. Weight-Related Outcomes

Weight-related outcomes included weight status based on self-reported BMI and UWCB. To assess the weight status, participants were requested to provide their height and weight. After calculating their BMI (i.e., dividing the weight in kilograms by the square of their height in meters), they were classified based on the definition of obesity suggested by Korean Society for the Study of Obesity [37]. Participants were classified according to the percentile of BMI by their sex and age: “obese” (i.e., ≥95th percentile) and “non-obese” (i.e., <95th percentile). 

We measured UWCB by asking if at least one of the following methods was used for weight control in the past 30 days: (a) fasting for ≥24 h, (b) reduced food intake, (c) restricting one’s diet to a specific food (e.g., egg, milk, and grapes), (d) vomiting after having meals, (e) using non-prescribed weight-loss pills, (f) taking diuretics or laxatives, and (g) consuming weight-loss supplements. The possible responses were “yes” and “no.”

### 2.3. Data Analysis

We performed four phases of data analysis using SAS version 9.4: identifying (a) sample characteristics, (b) sex-specific LCMs of OBs, (c) significant predictors of the LCM, and (d) class-specific distribution estimates for weight-related outcomes by sex. First, we used descriptive statistics (i.e., frequency and weighted percentages) of the sample characteristics by sex. 

Second, to investigate sex-specific LCMs of OBs, we conducted a multiple-group LCA using eight OBs [21]. Following the identification of the best fitted latent class model using the entire sample, we estimated a latent class model with freely estimated parameters and another with equally constrained item-response probabilities across sex. This helped us confirm the establishment of measurement invariance [21,38]. While performing the likelihood-ratio difference test to examine differences in the *G*^2^s with degrees of freedom for each model, a significant *p*-value indicated that the measurement invariance should be rejected. In other words, latent class models should be separately estimated by sex [38]. To select the optimal number of latent classes, we successively estimated 1- to 5-class models. The accurate number of classes were selected based on model fit indices, model identification, parsimony, and model interpretability. In relation to model fit indices, higher entropy and lower likelihood-ratio statistic (*G*^2^), the Akaike information criterion (AIC), Bayesian information criterion (BIC), adjusted BIC, and log-likelihood suggested a better fitted model [21]. Higher percentage of seeds associated with the best fitted model indicated better model identification [38]. In addition, a simpler and theoretically interpretable model was preferable [21]. 

Third, we performed an LCA with covariates to identify characteristics predicting sex-specific LCMs. We included seven categorical factors dummy coded in the multinomial logistic regression analysis. A *p*-value less than 0.050 indicates that the distribution of LCM differed across the covariate of interest [21]. Fourth, we investigated class differences in two weight-related outcomes (i.e., weight status and UWCB) using the Bolck, Croon, and Hagenaars (BCH) approach for LCA with a distal outcome [39,40]. The BCH method, one of the three-step approaches, is advantageous in calculating more accurate distal outcome estimates than merely using posterior probabilities. This is because the aforementioned method takes into account uncertainty owing to the possibility of misclassification [39]. After estimating class-conditional probabilities of the distal outcomes, we conducted a separate Wald test to compare them between each pair of latent classes [39].

## 3. Results

### 3.1. Sample Characteristics

The total 57,303 adolescents comprised 29,841 and 27,462 boys and girls, respectively. Of these adolescents, 13.8% boys and 8.2% girls had obesity. Moreover, 26.0% and 46.8% boys and girls engaged in UWCB, respectively. Table 1 summarizes the sample characteristics by sex.

### 3.2. Multiple-Group LCA

Before identifying sex-specific LCMs, we estimated a latent class model using the entire sample to determine the establishment of measurement invariance according to sex. *G*^2^, AIC, BIC, and adjusted BIC continued to decrease with an increase in the number of latent classes (Table 2). However, the model identification, parsimony, and entropy values indicated that the 3-or 4-class models were most suitable. We selected the 3-class model considering a small change in the model fit indices between the aforementioned models, a higher entropy value, and higher model identification in 3-class model than 4-class model. Moreover, the 3-class model was more parsimonious and theoretically easier to interpret. 

To assess measurement invariance across sex, the likelihood-ratio difference test was performed between the constrained model and the unconstrained model. *G*^2^ values in the unconstrained and constrained models were 1871.18 (*df* = 459) and 4359.98 (*df* = 483), respectively. The result indicated that measurement invariance across sex was not established (Δ*G*^2^ = 2488.80, *df* = 24, *p* < 0.001); thus, sex-specific latent class models were estimated by separating data of boys and girls [38].

### 3.3. Sex-Specific LCMs of Obesogenic Behaviors in Korean Adolescents

In both samples, the 3-or 4-class models were suitable for the data. We eventually selected the 3-class model because of relatively small differences in model indices between 3- and 4-class models, higher entropy value and higher model identification, and its simplicity and easy interpretation (Table 2). 

The 3-class models were presented: mostly healthy behaviors (MH), poor dietary habits and high Internet use (PDHI), and poor dietary habits and substance use (PDSU) in boys and girls (Table 3). In both sexes, the MH class was the largest group (53.9% and 47.9% of boys and girls, respectively), followed by PDHI (35.4% and 44.8%) and PDSU (10.8% and 7.4%). Overall, the probabilities of non-daily fruit and vegetable consumption in all classes were higher than 85.0%. The MH class was the least likely to engage in all OBs, compared to other classes. Compared to the MH class, adolescents belonging to the PDHI class displayed higher probabilities of skipping breakfast, SSB and fast food intake, non-daily fruit and vegetable consumption, and NAIU. Particularly, the PDHI class demonstrated the highest probability of consuming SSB and using the Internet for non-academic purposes compared to other classes. The probability of consuming SSB three or more days a week was 96.6% and 79.5% for boys and girls, respectively. The probability of NAIU for two or more hours was 51.2% and 60.5% for boys and girls, respectively. Those in the PDSU class displayed highest probabilities of engagement in breakfast skipping, non-daily fruit and vegetable consumption, and monthly smoking and drinking. The PDSU classes by sex were distinctly separated by their monthly substance use characteristics. Specifically, boys in the PDSU class exhibited higher probability of monthly tobacco product use than girls (69.2% and 50.3% of boys and girls, respectively). In addition, the probabilities of fast food consumption were less than 50.0% in all classes, but more than three times higher among those in the PDHI and PDSU classes than those in the MH class.

### 3.4. Predictors of LCM

To investigate the predictors of LCM, we used the MH class as a reference (Table 4). For both sexes, school year, area of residence, perceived academic performance, stress, depressive feeling, and sleep satisfaction significantly predicted the LCMs. Moreover, perceived economic status predicted the LCM in girls. 

The risk of being in the PDSU class was significantly higher in high school students than in middle school students (Odds ratios (ORs) = 5.72 and 3.20 for boys and girls, respectively). However, the risk of being in the PDHI class for boys was higher in middle school students than in high school students (OR = 0.86). Among both sexes, those living in suburban or rural areas were at a higher risk of belonging to the PDSU class than their urban counterparts (ORs = 0.82 and 0.75 for boys and girls, respectively). In girls, lower perceived economic status significantly increased the risk of belonging to the PDSU class, compared to the MH class (OR = 1.47). For both sexes, lower perceived academic performance and higher stress, depressive feelings, and sleep dissatisfaction were associated with an increased risk of being in the PDHI and PDSU classes, compared to the MH class. In addition, the three psychological predictors associated with the PDHI and PDSU classes in both sexes generally displayed stronger associations with the PDSU class than the PDHI class.

### 3.5. Class Difference in Weight-Related Outcomes

**Figure 1:**
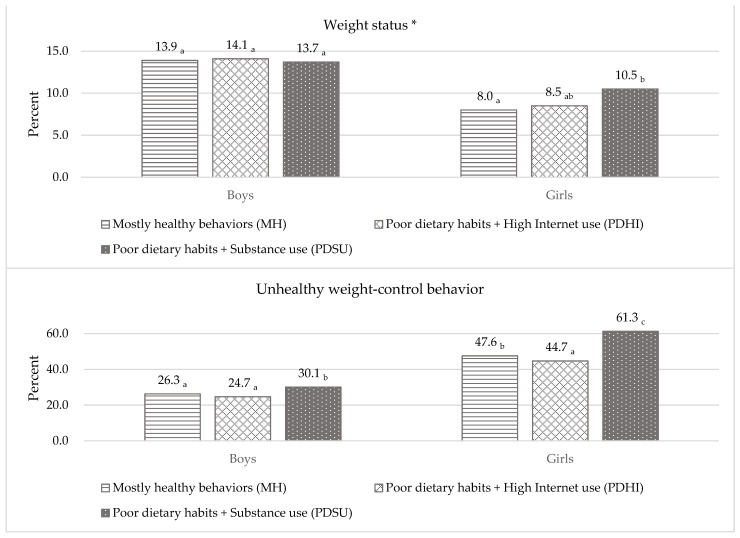
Proportion of distal outcomes by latent class (*n* = 57,303). Note. Estimates with the different subscripted letter indicate statistically significant differences at the 0.050 alpha level. * Indicates the proportion of obese participants.

Sex-specific classes of OBs were compared on their weight-related outcomes (Figure 1). The prevalence of obesity in all classes did not significantly differ among boys. In girls, the PDSU class reported on the highest proportion of obesity, followed by the PDHI class; however, the difference was insignificant. The PDSU class reported on a significantly higher rate of obesity than the MH class (*p* = 0.012). There was no significant difference in the proportion of obesity between the MH and PDHI classes. 

Regarding the proportion of UWCB, the PDSU class reported on the highest among all classes in boys. Specifically, the PDSU class reported on a significantly higher rate of UWCB compared to the MH class (*p* < 0.001) and PDHI class (*p* < 0.001). There was no significant difference in the proportion of UWCB between the MH and PDHI classes among boys. In girls, the PDSU class displayed the highest proportion of UWCB, followed by the MH and the PDHI classes. The PDSU class had significantly higher UWCB compared to the MH class (*p* < 0.001) and PDHI class (*p* < 0.001). Interestingly, the MH class reported on significantly higher rate of UWCB engagement, compared to the PDHI class (*p* = 0.009).

## 4. Discussion

This study demonstrated sex-specific latent class models of adolescent OBs. For both sexes, school year, residential area, perceived academic performance, stress, depressive feeling, and sleep satisfaction significantly predicted the LCMs. Moreover, perceived economic status predicted the LCM among girls. We also found differences in sex-specific LCMs in proportions of obesity and UWCB. 

### 4.1. Sex-Specific LCMs of Obesogenic Behaviors

We identified three classes across sex, namely the MH, PDHI, and PDSU classes. Previous studies on sex-specific patterns of adolescent OBs identified three to five latent classes: (a) the healthy class with high probabilities of healthy dietary habits and adequate physical activities, (b) the physically active class with high probabilities of poor dietary habits, (c) the sedentary class with high probabilities of poor dietary habits, and (d) the substance use class with high probability of poor dietary habits and moderate physical activities [20,23]. Despite the need for cautiously comparing LCMs between studies owing to different OBs included in the LCA and the limited number of studies examining sex-specific LCMs of adolescent OBs, our results were consistent with previous studies [20,23]. OB patterns were divided into classes with the lowest likelihood of OBs, with moderate OBs, and with the most severe OBs. In addition, poor dietary habits were common features of all classes [20,23,24]. Particularly, despite measuring “daily” and “non-daily” fruit and vegetable intake using criteria less stringent than the nutritional guidelines suggested by Korean Ministry of Health and Welfare [30], we observed high probabilities of non-daily fruit and vegetable consumption across all classes. This result is not surprising, considering the previous evidence from studies conducted in the U.S. and Canada [23,24] and the literature describing poor dietary habits in adolescents [41].

In our study, the distribution and characteristics of OB patterns overlapped between boys and girls. Nonetheless, some characteristics of OB patterns demonstrated differences by sex. For example, the prevalence and characteristics of PDSU differed by sex. Specifically, the prevalence of the PDSU class and their probability of monthly tobacco product use were considerably higher for boys than for girls, consistent with a recent study [20]. This result may be attributed to a higher rate of substance use in boys than girls, particularly smoking behaviors [4,42]. Our results are also supported by Fleary [20] who suggested that multiple OBs, including health promoting and risk behaviors, occur on a sex-specific continuum. Thus, the efforts to prevent and improve adolescent weight-related outcomes should be preceded by a better understanding of the multidimensional and complex characteristics of sex-specific OB patterns. 

### 4.2. Predictors of Latent Class Membership

Some demographic characteristics included as covariates distinguished the class memberships across sex. For example, among boys, high school students are less likely to be in the PDHI class than the MH class. This finding may be partly attributed to the relationship between Internet use and the age and sex among Korean adolescents. The risk of NAIU is higher in boys and middle school students than in girls and high school students [43]. In contrast to boys, girls who perceived lower economic status demonstrated a greater risk of belonging to the PDSU class than those who perceived middle or high economic status. This finding is consistent with the literature [35]. This may be attributed to the engagement of parents or family members of lower socioeconomic status in OBs, which may contribute to adolescent OBs (i.e., modeling), particularly in girls [35]. This phenomenon indicates that the social status of the family is one of important predictors affecting adolescent OBs.

Several other demographic characteristics significantly predicted LCMs for both sexes, in accordance with the existing literature. For example, high school students were more likely to be in the PDSU class than middle school students [25,44]. This finding may be attributed to the increase in exposure to obesogenic environments (e.g., peer social pressure for poor dietary habits, greater accessibility to substances) with increasing age [45,46,47]. In addition, the risk of belonging to the PDSU class was significantly higher in suburban or rural students than in urban students. Considering that the residential area did not significantly predict the risk of belonging to the PDHI class, this finding may be partly attributed to higher rates of tobacco product and alcohol use in suburban or rural students compared to their urban counterparts [33,34,48]. The literature indicated that regional characteristics contribute to the difference in substance use among adolescents [49,50]. In rural communities, while social norms and regulations on adolescent substance use are weak, access to substances is relatively easy [49,50]. 

Consistent with previous studies, the lower the academic performance [36] and the poorer the psychological status [31,32,51], the greater the risk of belonging to the class with higher probabilities of OBs. Those with a greater level of stress, depressive feelings, and sleep dissatisfaction tended to display stronger associations with the PDSU class than the PDHI class. OBs in adolescence have bidirectional relationships with psychological status. Those with poorer psychological status are more likely to engage in screen-based activities and substance use to alleviate stressful and depressive feelings [31,52]. Inadequate nutrition consumption and substance use in adolescence may cause difficulties in normal brain function and mood regulation [53,54]. Moreover, sufficient sleep may not be a priority for adolescents who engage in substance use with their peers [55]. Considering the high vulnerability to psychological problems and negative prospects for OBs in adolescence, those in the PDSU class are a critical subgroup with respect to their OBs and poor psychological status. In addition, those with psychological symptoms may be a target group for early prevention of OBs.

### 4.3. Class Difference in Weight-Related Outcomes

Considering the class differences in the proportion of obesity and UWCB, we identified distinct characteristics of sex-specific LCMs of OBs. For example, while there was no significant difference in obesity rates across the latent classes for boys, the obesity rate was the highest in the PDSU class for girls, consistent with previous studies [18,23]. There are two potential reasons for the weakened relationship between obesity and LCM in boys. Boys generally increase muscle mass for a masculine body shape [56] and spend more time in various physical activities than girls [57]. 

We also found that the PDSU class demonstrated higher UWCB prevalence than other classes, thereby suggesting dietary habits and substance use are robust predictors of UWCB in adolescence. This phenomenon could be attributed to lower body satisfaction in the class characterized by a higher probability of sedentary behavior and poor dietary habits [23]. According to the problem behavior theory, health risk behaviors in adolescence co-occur and co-vary owing to common determinants [58]. Both UWCB and substance use are associated with impulsivity-like traits [59,60]. Substance users are more likely to participate in UWCB requiring relative little effort for rapid weight control, despite the detrimental consequences of UWCB [59].

In addition, class memberships in girls were approximately twice likely to adopt UWCB than boys, thus suggesting girls are more enthusiastic about losing weight [14]. This is because girls are more likely to have greater body dissatisfaction than boys [23] and are more likely to adopt UWCB to achieve a slender physique that is considered ideal socio-culturally [56].

An unexpected finding was the substantial proportion of UWCB in the MH class. Despite the lowest obesity rate in the MH class for girls, approximately half of them engaged in the UWCB. Similarly, a recent study reported that the proportion of UWCB in the healthy class was lower than that in the severely unhealthy class; however, it was higher than that in the moderately unhealthy classes [20]. This phenomenon may be partly attributed to the fact that despite the lower obesity prevalence in the healthy class compared to other classes, body satisfaction was not significantly higher in the healthy class than in other classes [23]. Moreover, normal-weight girls tend to overestimate their own weight [61]. Therefore, health professionals should not consider the class characterized by healthy behavior as a low-risk group of weight-related outcomes. Instead, they should consider not only distinct OB patterns but also sex-specific associations between each OB pattern and weight-related outcomes.

### 4.4. Limitations

The results of this study should be cautiously interpreted in consideration of potential limitations. First, we could not determine the causal relationship between sex-specific LCMs and weight-related outcomes because of cross-sectional data. Second, the BMI was based on self-reported weight and height of the participants. However, BMI based on self-reported parameters accurately differentiates obese adolescents [62]. Third, as predictors of sex-specific LCMs, we did not include developmental and environmental characteristics (e.g., impulsivity, obesogenic home environment, and peer pressure on OBs) that affected weight-related outcomes [46,47,60].

## 5. Conclusions

This study identified the 3-class models of OBs in boys and girls using LCA, namely the MH, PDHI, and PDSU classes. We found sex-specific findings on the distribution and characteristics of the three classes and the association of sex-specific LCMs with potential predictors. Rather than focusing on a single OB, health professionals should develop interventions tailored to sex-specific adolescent OB patterns [20]. This study extended previous research on adolescent OBs by examining the relationship between sex-specific latent classes and weight-related outcomes. Classes with low probabilities of OB do not necessarily have better weight-related outcomes. This necessitates providing interventions that precisely target each latent class while considering differences in its association with weight-related outcomes. For example, interventions for boys and girls in the PDSU class with a high prevalence of UWCB should not only target multiple modifiable OBs but also include healthy weight-control methods. In addition, interventions for girls in the MH class with a substantial prevalence of UWCB should include healthy weight-control methods despite their low likelihood of OBs and obesity.

Future research on adolescent OBs should consider the following issues. First, longitudinal studies are required to establish causal relationships between OB patterns and later weight-related outcomes in adolescence. Second, as this is the first study to examine the association between latent classes of OBs and UWCB, repeated studies on class differences in UWCB among adolescents from different cultures are warranted. Third, further studies should include a wider variety of potentially associated factors (e.g., family, school, and peer environments, developmental characteristics, and weight-related concerns) to refine the characteristics of adolescent OB patterns [13].

## Figures and Tables

**Table 1:** Sample characteristics (*n* = 57,303).

Factor	Frequency (%) *			
	Boys (*n* = 29,841)		Girls (*n* = 27,462)	
School year				
Middle school	15,401	(47.7)	13,983	(48.1)
High school	14,440	(52.3)	13,479	(51.9)
Area of residence				
Suburban or rural	14,475	(49.4)	13,472	(49.2)
Urban	15,366	(50.6)	13,990	(50.8)
Perceived economic status				
High or middle	26,146	(87.8)	23,816	(87.2)
Low	3695	(12.3)	3646	(12.9)
Perceived academic performance				
High or middle	20,530	(68.5)	18,647	(67.9)
Low	9311	(31.5)	8815	(32.1)
Stress				
Low or average	20,446	(68.3)	14,079	(51.2)
High	9395	(31.7)	13,383	(48.8)
Depressive feeling				
No	23,346	(77.8)	17,929	(65.4)
Yes	6495	(22.2)	9533	(34.6)
Sleep satisfaction				
Satisfied or average	18,610	(61.4)	12,621	(45.2)
Dissatisfied	11,231	(38.6)	14,841	(54.8)
Breakfast skipping				
>2 days/week	14,321	(48.1)	14,662	(52.9)
≤2 days/week	15,520	(52.0)	12,800	(47.1)
Sugar-sweetened beverage intake				
≥3 days/week	19,650	(66.2)	15,242	(55.5)
<3 days/week	10,191	(33.8)	12,220	(44.5)
Fast food consumption				
≥3 times/week	8011	(27.5)	6381	(23.4)
<3 times/week	21,830	(72.5)	21,081	(76.6)
Fruit and vegetable consumption				
Non-daily	26,109	(87.4)	24,393	(88.6)
Daily	3732	(12.6)	3069	(11.4)
Total physical activity				
≥60 min/day	26,814	(90.5)	26,746	(97.4)
<60 min/day	3027	(9.6)	716	(2.6)
Non-academic Internet use				
≥2 h/day	13,464	(45.1)	14,445	(52.7)
<2 h/day	16,377	(54.9)	13,017	(47.3)
Tobacco product use				
No	26,952	(89.7)	26,323	(95.9)
Yes	2889	(10.3)	1139	(4.1)
Alcohol use				
No	25,000	(83.2)	23,903	(87.0)
Yes	4841	(16.9)	3559	(13.0)
Weight status				
Non-obese	25,004	(86.2)	24,439	(91.9)
Obese	4055	(13.8)	2250	(8.2)
Unhealthy weight control behaviors				
No	22,046	(74.0)	14,464	(53.3)
Yes	7795	(26.0)	12,998	(46.8)

* Unweighted frequency and weighted percentage.

**Table 2:** Fit statistics of latent classes of obesogenic behaviors among boys and girls (*n* = 57,303).

Number of Latent Classes	*G* ^2^	Degree of Freedom	AIC	BIC	Adjusted BIC	Entropy	Log-Likelihood	Percentage of Seeds Associated with Best Fitted Model
Total								
1	13,719.60	247	13,735.60	13,807.25	13,781.83	1.00	−223,225.48	100.0
2	5055.70	238	5089.70	5241.96	5187.93	0.75	−218,893.54	100.0
**3**	**1841.57**	**229**	**1893.57**	**2126.43**	**2043.80**	**0.51**	**−217,286.47**	**100.0**
4	842.81	220	912.81	1226.27	1115.04	0.48	−216,787.09	50.0
5	591.24	211	679.24	1073.31	933.48	0.54	−216,661.30	15.0
Boys								
1	7686.18	247	7702.18	7768.61	7743.18	1.00	−121,481.34	100.0
2	2667.31	238	2701.31	2842.47	2788.45	0.72	−118,971.90	100.0
**3**	**1135.00**	**229**	**1187.00**	**1402.89**	**1320.26**	**0.54**	**−118,205.74**	**100.0**
4	585.64	220	655.64	946.27	835.04	0.51	−117,931.06	45.0
5	411.37	211	499.37	864.73	724.90	0.55	−117,843.93	20.0
Girls								
1	6044.51	247	6060.51	6126.28	6100.85	1.00	−99,990.15	100.0
2	2554.12	238	2588.12	2727.87	2673.84	0.80	−98,244.95	20.0
**3**	**736.18**	**229**	**788.18**	**1001.92**	**919.29**	**0.53**	**−97,335.98**	**100.0**
4	371.60	220	441.60	729.32	618.09	0.45	−97,153.69	95.0
5	286.48	211	374.48	736.19	596.35	0.48	−97,111.13	5.0

Note. Bold letters indicate the best fitting models. *G*^2^ = the likelihood-ratio statistic; AIC = Akaike’s information criterion; BIC = Bayesian information criterion.

**Table 3:** Item-response probabilities of obesogenic behaviors among boys and girls (*n* = 57,303).

	Boys (*n* = 29,841)			Girls (*n* = 27,462)		
	Mostly Healthy Behaviors (MH)	Poor Dietary Habits + High Internet Use (PDHI)	Poor Dietary Habits + Substance Use (PDSU)	Mostly Healthy Behaviors (MH)	Poor Dietary Habits + High Internet Use (PDHI)	Poor Dietary Habits + Substance Use (PDSU)
Probability of membership	0.539	0.354	0.108	0.479	0.448	0.074
Breakfast skipping (>2 days/wk)	0.431	**0.506**	**0.641**	0.442	**0.597**	**0.751**
SSB intake (≥3 days/wk)	0.429	**0.966**	**0.797**	0.300	**0.795**	**0.754**
Fast food consumption (≥3 days/wk)	0.104	0.476	0.409	0.034	0.415	0.413
Fruit and vegetable consumption (non-daily)	**0.866**	**0.873**	**0.924**	**0.857**	**0.914**	**0.936**
Total physical activity (non-daily)	0.091	0.115	0.113	0.028	0.022	0.038
Non-academic Internet use (≥2 h/day)	0.408	**0.512**	0.469	0.457	**0.605**	0.491
Smoking during the past 30 days (Yes)	0.018	0.036	**0.692**	0.004	0.005	**0.503**
Drinking during the past 30 days (Yes)	0.067	0.112	**0.801**	0.067	0.079	**0.845**

Note. Bold figures indicate that the item-response probability is 0.500 or above. SSB = sugar-sweetened beverage.

**Table 4:** Predictors of latent class membership among boys and girls (*n* = 57,303).

Predictor	Boys (*n* = 29,841)					Girls (*n* = 27,462)				
	*p*-Value	Poor Dietary Habits + High Internet Use (PDHI)		Poor Dietary Habits + Substance Use (PDSU)		*p*-Value	Poor Dietary Habits + High Internet Use (PDHI)		Poor Dietary Habits + Substance Use (PDSU)	
		OR	95% CI	OR	95% CI		OR	95% CI	OR	95% CI
School year										
High school (ref. = middle school)	<0.001	0.86	(0.79, 0.95)	5.72	(5.03, 6.50)	<0.001	1.00	(0.91, 1.09)	3.20	(2.82, 3.64)
Area of residence										
Urban area (ref. = suburban/rural area)	<0.001	1.02	(0.93, 1.11)	0.82	(0.74, 0.89)	<0.001	0.96	(0.88, 1.05)	0.75	(0.68, 0.83)
Perceived economic status										
Low (ref. = high or middle)	0.052	0.94	(0.82, 1.08)	1.11	(0.97, 1.26)	<0.001	1.11	(0.97, 1.28)	1.47	(1.28, 1.69)
Perceived academic performance										
Low (ref. = high or middle)	<0.001	1.62	(1.47, 1.80)	2.49	(2.25, 2.76)	<0.001	1.80	(1.62, 1.99)	2.53	(2.26, 2.84)
Stress										
High (ref. = low or average)	<0.001	1.22	(1.10, 1.34)	1.18	(1.07, 1.31)	<0.001	1.52	(1.39, 1.67)	1.71	(1.53, 1.91)
Depressive feeling										
Yes (ref. = no)	<0.001	1.53	(1.36, 1.72)	2.80	(2.50, 3.14)	<0.001	1.56	(1.41, 1.72)	3.36	(2.99, 3.77)
Sleep satisfaction										
Dissatisfied (ref. = average or satisfied)	<0.001	1.54	(1.41, 1.69)	1.89	(1.71, 2.09)	<0.001	1.36	(1.24, 1.48)	1.57	(1.41, 1.75)

Note. The reference group = mostly healthy behaviors (MH). OR = odds ratio; CI = confidence interval; ref. = reference.

## Data Availability

No new data were created or analyzed in this study. Data sharing is not applicable to this article.

## Abbreviations

OB: obesogenic behaviors; UWCB: Unhealthy weight-control behavior; LCA: latent class analysis; LCM: latent class membership; MH: mostly healthy behaviors; PDHI: poor dietary habits and high Internet use; PDSU: poor dietary habits and substance use; NAIU: non-academic Internet use; OR: Odds ratio.
